# The Paradox of Belonging: Minority Stress, Community Belongingness, and Subjective Well-Being Among Black LGBTQ+ Adults

**DOI:** 10.3390/bs15121604

**Published:** 2025-11-21

**Authors:** Miya C. Tate, Shawndaya S. Thrasher, Keith J. Watts, Janet K. Otachi, DeKeitra Griffin, Justin X. Moore

**Affiliations:** 1 School of Social Work, University of Georgia, Athens, GA 30602, USA; miya.tate@uga.edu; 2 School of Social Work, Louisiana State University, Baton Rouge, LA 70802, USA; sthrasher@lsu.edu (S.S.T.); dgrif36@lsu.edu (D.G.); 3 College of Social Work, University of Kentucky, Lexington, KY 40508, USA; 4 Kent School of Social Work and Family Science, University of Louisville, Louisville, KY 40292, USA; janet.otachi@louisville.edu; 5 College of Medicine, University of Kentucky, Lexington, KY 40506, USA; jx.moore@uky.edu

**Keywords:** Black LGBTQ+, minority stress, community belongingness, well-being, intersectionality, health disparities, resilience

## Abstract

Black LGBTQ+ adults face significant health disparities stemming from intersecting minority stressors. While community belongingness is often theorized as a protective factor, these communities can also be sites of exclusion, creating a complex dynamic. This study’s objective was to test whether community belongingness moderates the relationship between minority stress, operationalized as microaggressions, and subjective well-being in a national sample of Black LGBTQ+ adults. Data were taken from a national online survey of 345 Black LGBTQ+ adults conducted between November 2020 and January 2021. We used validated scales to measure experiences of microaggressions, subjective well-being, and community belongingness. A moderated multiple regression analysis was conducted using the PROCESS macro to test for an interaction effect, controlling for demographic covariates. Minority stress was significantly negatively associated with well-being (b = −0.11, *p* = 0.005), while community belongingness was positively associated with well-being (b = 0.43, *p* < 0.001). A significant interaction emerged (b = −0.01, *p* = 0.021). Simple slopes analysis revealed that the negative relationship between minority stress and well-being was strongest for individuals with high community belongingness (b = −0.18, *p* < 0.001) and was not significant for those with low belongingness, potentially indicating an exacerbating effect. Contrary to the buffering hypothesis, community belongingness paradoxically amplified the negative impact of minority stress on well-being. This paradox of belonging suggests that highly connected communities may become sites for trauma bonding, which can amplify distress. Public health efforts must focus not only on fostering connection but on building communities that are resourceful, inclusive, and capable of transforming shared experiences into collective empowerment.

## 1. Introduction

Racial, sexual, and gender discrimination are critical public health challenges that create significant health disparities, particularly for Black LGBTQ+ (lesbian, gay, bisexual, transgender, queer, and other sexual and gender minorities) adults (Choi et al., 2021; English et al., 2021; Meyer, 2003; Page et al., 2022). Scholarship has moved beyond viewing these biases as individual stressors to understanding them as forms of intersecting structural oppression, where state-level policies and systemic racism synergistically harm the health of Black sexual minority individuals in ways not seen for their white peers (English et al., 2021). This creates a “double marginalization” for Black LGBTQ+ people, who navigate anti-Black racism within predominantly white LGBTQ+ spaces and, simultaneously, heterosexism and transphobia within some Black community contexts (Page et al., 2022; Sadika et al., 2020). The persistent stress arising from this dual marginalization contributes to a higher prevalence of adverse health outcomes, including depression and barriers to accessing affirming healthcare (Choi et al., 2021; Institute of Medicine, 2011).

While extensive research documents the negative impacts of discrimination on mental health (Meyer, 2003; Sadika et al., 2020; Truong et al., 2020), fewer studies have focused on factors promoting resilience and well-being among multiple marginalized populations, such as Black LGBTQ+ populations. Specifically, a critical gap exists in understanding the agentic strategies Black LGBTQ+ people use to build their own communities as a form of resistance (Page et al., 2022; Rosenberg, 2021) and cultivate social capital—the resources afforded by social connections—as a protective asset against systemic harm (Husbands et al., 2022; Zarwell et al., 2021). However, recent literature also complicates this picture, revealing that mainstream LGBTQ+ communities are often sites of exclusion, racism, and community policing that create barriers to belonging for their most marginalized members (McCormick & Barthelemy, 2021; Parmenter et al., 2021b). This central tension—between community as a site of both potential harm and profound resilience—animates the present study. Further, recent quantitative work demonstrates that the type of community matters immensely; belongingness to the Black community was uniquely predictive of better mental health, while belongingness to the Black LGBTQ+ community was uniquely predictive of higher well-being (Watts & Thrasher, 2023). Building on that knowledge, the present study moves beyond direct effects to test a more nuanced question: how does community belongingness interact with minority stress to influence the well-being of Black LGBTQ+ adults? This study is uniquely situated during the dual pandemics of COVID-19 and a national reckoning with racial injustice, providing a critical snapshot of these dynamics under heightened societal stress.

Three complementary theoretical frameworks help explain these health inequities and potential protective factors. First, minority stress theory (Meyer, 1995, 2003) posits that stigmatized minorities experience unique, chronic stressors related to their marginalized status. These stressors can be distal (e.g., overt discrimination, systemic inequalities) or proximal (e.g., internalized homophobia/transphobia, expectations of rejection). This chronic stress contributes to adverse mental and physical health outcomes. Second, intersectionality theory (Crenshaw, 1989) extends this understanding by recognizing that multiple social identities (e.g., race, gender, sexual orientation) do not simply add to one another but intersect to create unique experiences of oppression and privilege. For Black LGBTQ+ individuals, the intersection of racial and sexual, and gender minority status creates distinct stressors that are qualitatively different from those experienced by individuals with only one of these identities. Third, belongingness theory suggests that connection to affirming, identity-based communities may be a critical protective mechanism against these stressors. This theory posits that belonging is a fundamental human need essential for psychological well-being (Baumeister & Leary, 1995). For multiply marginalized populations, connection to an affirming, identity-based community is a primary site for fulfilling this need. Grounded in a multidimensional framework of collective identity, we conceptualize this connection—specifically to the Black LGBTQ+ community—through key dimensions such as affective attachment (an emotional bond to the group) and social embeddedness (integration into social networks), which provide individuals with social support and resources (in essence, social capital) for coping with discrimination and marginalization (Ashmore et al., 2004; Zarwell et al., 2021). By integrating these theoretical insights, the present study aims to provide a nuanced understanding of whether community belongingness can moderate the relationship between minority stress and subjective well-being.

Existing literature underscores the profound impact of minority stress on mental health outcomes among marginalized populations. Yet, there is a notable gap in research addressing whether community belongingness may mitigate these effects among Black LGBTQ+ individuals. However, while many studies affirm the direct, positive role of community belonging (Moore et al., 2021; Roberts & Christens, 2021), less have empirically tested its function as a statistical moderator of the minority stress process specifically among Black LGBTQ+ individuals, representing a critical gap in the literature. Prior investigations have demonstrated that microaggressions and other forms of discrimination are closely linked to lower levels of subjective well-being (Ashmore et al., 2004; Balsam et al., 2011). Furthermore, a substantial body of prior research suggests that belonging to an affirming community can serve as a protective buffer, providing health-promoting strengths and fostering resilience that reduces the psychological toll of these negative experiences (Barr et al., 2016; DeMarco & Newheiser, 2019; Hailey et al., 2020; Hudson & Romanelli, 2020; Quinn et al., 2023). This study, therefore, extends current scholarship by focusing on the unique intersectional experiences of Black LGBTQ+ adults, investigating the relationship between minority stress and subjective well-being, and whether this relationship is moderated by community belongingness among this marginalized group. Accordingly, this study tested two primary hypotheses:

1. Experiences of minority stress will be negatively associated with subjective well-being.
2. Community belongingness will moderate this relationship, such that the negative association between minority stress and subjective well-being will be weaker for individuals with higher levels of community belongingness.

## 2. Materials and Methods

This study employed a cross-sectional, correlational design. Data were derived from a one-time national online survey of 345 Black LGBTQ+ adults between November 2020 and January 2021. We employed purposive and snowball sampling techniques to reach this hidden and marginalized population (McConnell et al., 2018). Inclusion criteria required participants to be 18 years or older, identify as both Black and LGBTQ+, reside in the United States, and be able to complete an English-language survey. Participants were recruited through multiple channels to ensure diverse representation within the Black LGBTQ+ community. Electronic recruitment materials were distributed through LGBTQ+ community organizations, social media platforms (i.e., Facebook, Instagram, Twitter), and identity-based professional networks. This multi-pronged strategy was intended to capture a more representative sample than a single recruitment source would allow. For instance, recruiting on social media platforms can enhance the effectiveness of reaching younger and more geographically dispersed adults, while partnerships with specific community organizations and professional networks (e.g., those for Black social workers, academics, or health professionals) can help engage a wider range of age groups, educational backgrounds, and socioeconomic statuses, thus enhancing the sample’s overall diversity.

Data were collected through a secure web-based survey using REDCap (Research Electronic Data Capture), a HIPAA-compliant platform (Harris et al., 2009). Participants were offered the option of being included in a one-time random drawing for one of twenty-five $25 gift cards. The study protocol was approved by the Institutional Review Board of Virginia Commonwealth University (HM20020418). All participants were presented with a detailed study information sheet and provided electronic informed consent before beginning the survey.

### 2.1. Measures

Subjective Well-being was assessed using the 14-item Mental Health Continuum—Short Form (MHC-SF; α = 0.893) (Keyes, 2002, 2005). This validated instrument measures emotional well-being (3 items), psychological well-being (6 items), and social well-being (5 items) experienced during the previous two weeks. Sample items included “During the past two weeks, how often did you feel interested in life?” and “During the past two weeks, how often did you feel that you belonged to a community?” Responses were recorded on a 7-point Likert scale ranging from 0 (never) to 6 (every day). All 14 items were summed to produce a total subjective well-being score (possible range: 0–84), with higher scores indicating greater subjective well-being. The MHC-SF has demonstrated strong psychometric properties across diverse populations, including racial and sexual minorities (Diener et al., 2018).

Minority Stress was operationalized as experiences of microaggressions and measured using the 18-item LGBT People of Color Microaggressions Scale (α = 0.882) (Balsam et al., 2011). This measure was designed to capture people’s unique experiences with intersecting racial, sexual, and gender minority identities. The scale assessed both the frequency and emotional impact of microaggression experiences using a 5-point Likert scale (0 = “Did not happen/not applicable to me” to 5 = “It happened, and it bothered me EXTREMELY”). Sample items included “White LGBT people saying things that are racist”, “Not being accepted by other people of your race/ethnicity because you are LGBT”, and “Difficulty finding friends who are LGBT and from your racial/ethnic background.” The 18 items were summed to create a total minority stress score (possible range: 0–90), with higher scores indicating more frequent and distressing microaggression experiences.

Community Belongingness was measured using 9 self-reported items (α = 0.861) adapted from the Transgender Community Belongingness Scale (Barr et al., 2016). Following Watts and Thrasher (2023), the wording was modified to reflect belongingness to Black LGBTQ+ communities specifically. Participants responded to items such as “I feel like a member of the Black LGBTQ community” and “I feel like a member of the Black LGBTQ+ community” on a 5-point Likert scale (1 = ‘not at all’, 5 = ‘all of the time’). The nine items were summed to produce a total community belongingness score (possible range: 9–45), with higher scores indicating a stronger sense of belonging to identity-based communities.

Covariates included age, income, and education. Age was measured by asking participants, “What is your current years of age?” ($\bar{x}$ = 27.95, SD = 6.36). Household income was collected as a categorical variable with 11 response options ranging from “less than $25,000” to “$150,000 or more”. Given the skewed distribution, the median income category of $45,000 to $54,999 was used to dichotomize income, specifically as 0 = $54,999 or less and 1 = $55,000 or more to represent those making at/below the median or higher. Based on the sample distribution, education was measured via eight categories and dichotomized (0 = some college or less and 1 = bachelor’s degree or higher). These demographic variables were selected as covariates based on their established associations with both minority stress and health outcomes in prior literature.

### 2.2. Analytic Plan

Pre-analysis screening procedures were conducted to ensure data met assumptions for regression analysis. This included assessing multicollinearity through correlation coefficients and variance inflation factors (VIF), with tolerance scores revealing no violation of the multicollinearity assumption (all VIF < 2.5) (Craney & Surles, 2002). A missing value analysis found that missingness ranged from 5 to 25% for primary variables in the study. To address missing data, Multiple Imputation by Chained Equations (MICE) with 10 imputations was employed to minimize bias and maximize statistical power. All parameter estimates and standard errors for subsequent regression models were computed on the combined imputed datasets using Rubin’s rules to ensure the accuracy of the final results.. A power analysis was performed and indicated the sample size provided adequate statistical power (>0.80) to detect small to medium effect sizes (f^2^ ≥ 0.05) with alpha set at 0.05. Descriptive statistics were calculated for all study variables. Next, bivariate correlation analysis assessed relationships between minority stress, community belongingness, and subjective well-being. Finally, a moderated regression analysis was conducted using PROCESS Macro in SPSS version 29 to determine the direct effect of minority stress on well-being and whether community belongingness moderated the minority stress and well-being relationship, controlling for age, income, and education. Furthermore, a simple slope analysis was conducted to interpret significant interactions (Aiken & West, 1991) using PROCESS Macro. All analyses were performed using SPSS version 29 (IBM Corp., Armonk, NY, USA). Statistical significance was established at *p* < 0.05 (two-tailed). It is important to note that PROCESS Macro, which reports only unstandardized regression coefficients, was used to interpret direct effects and moderation analyses.

## 3. Results

### 3.1. Descriptive Statistics

Participants reported moderately high levels of minority stress (M = 42.47, SD = 13.05), relatively high subjective well-being (M = 54.92, SD = 10.32), and moderate community belongingness (M = 30.39, SD = 6.02). The sample consisted of young to middle-aged adults with a mean age of 27.95 years (SD = 6.37, range = 18–64). Regarding education, most participants (72.17%) reported having at least a college degree. For income, 40.58% of participants reported an annual household income of $55,000 or more, while 59.42% reported incomes below this threshold (see Table 1).

### 3.2. Bivariate Correlation Analysis

Pearson correlation analysis (Table 2) revealed a significant negative relationship between minority stress and subjective well-being (*r* = −0.196, *p* < 0.001). Subjective well-being was positively correlated with community belongingness (*r* = 0.313, *p* < 0.001). The interaction between minority stress and community belongingness was negatively associated with subjective well-being (*r* = −0.221, *p* < 0.001), suggesting a potential moderating effect. Regarding covariates, age was positively correlated with well-being (*r* = 0.188, *p* < 0.001) and community belongingness (r = 0.170, *p* = 0.002) but not significantly correlated with minority stress (*r* = −0.079, *p* = 0.145). Education was positively correlated with community belongingness (*r* = 0.113, *p* = 0.035) but not significantly correlated with well-being (*r* = 0.029, *p* = 0.586) nor minority stress (*r* = −0.101, *p* = 0.060). Income was not significantly correlated with well-being (*r* = 0.026, *p* = 0.627), community belongingness (*r* = 0.068, *p* = 0.209), or minority stress (*r* = −0.033, *p* = 0.545).

### 3.3. Multivariate Linear Regression Analysis

**Figure 1:**
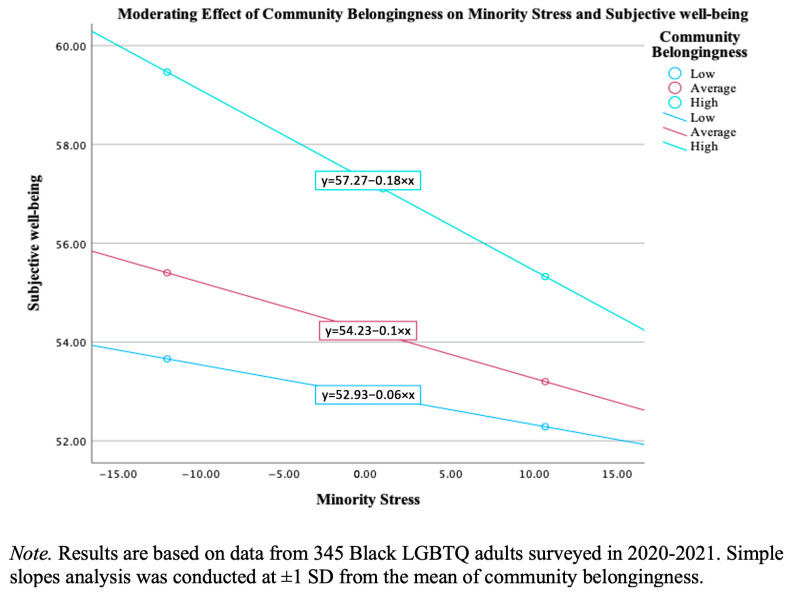
Simple slope visualization.

After controlling for age, income, and education, a multivariate linear regression (see Table 3) revealed that minority stress was significantly negatively associated with subjective well-being (b = −0.11, SE = 0.04, *p* = 0.005, 95% CI [0.19, 0.03]). Community belongingness demonstrated a statistically significant positive association with subjective well-being (b = 0.43, SE = 0.09, *p* < 0.001, 95% CI [0.26, 0.61]). The interaction between minority stress and community belongingness was statistically significant (b = −0.01, SE = 0.005, *p* = 0.021, 95% CI [0.02, 0.002]), indicating a moderating effect. To probe this interaction, a simple slopes analysis was performed (see Figure 1 for visualization and Table 4 for numerical results) at one standard deviation above and below the mean of community belongingness (Aiken & West, 1991). The analysis revealed that the negative relationship between minority stress and subjective well-being was significant and strongest for individuals with high levels of community belongingness (b = −0.18, *p* < 0.001). The relationship was also significant for those with average levels of belonging (b = −0.10, *p* = 0.023) but was not significant for those with low levels of community belongingness (b = −0.06, *p* = 0.23). This indicates that, contrary to a simple buffering effect, community belongingness appeared to exacerbate the negative impact of minority stress on well-being. The harmful effects of minority stress were most potent among individuals reporting the strongest community connections. Regarding covariates, age was statistically significantly associated with subjective well-being (b = 0.24, SE = 0.09, *p* = 0.007, 95% CI [0.06, 0.41]). Neither income (b = −0.58, SE = 1.08, *p* = 0.591, 95% CI [−2.70, 1.54]) nor education (b = −1.26, SE = 1.22, *p* = 0.302, 95% CI [−3.67, 1.14]) was a significant predictor of subjective well-being. The model was statistically significant (F(6, 338) = 10.66, *p* < 0.001) and accounted for 15.9% of the variance (R^2^ = 0.159), representing a medium size effect.

## 4. Discussion

This study examined the relationships between minority stress, community belongingness, and subjective well-being among a national sample of Black LGBTQ+ adults. Consistent with our first hypothesis and the foundational tenets of minority stress theory (Meyer, 1995, 2003), we found that greater experiences of microaggressions were significantly associated with lower subjective well-being. We also found that community belongingness, on its own, was a strong positive predictor of well-being. However, contrary to our second hypothesis that belongingness would serve as a protective buffer, our moderation analysis revealed a more complex and counterintuitive dynamic. The negative relationship between minority stress and well-being was statistically significant only for those with average and high levels of community belongingness, and was strongest for those with the highest reported sense of belonging. This finding suggests that under conditions of high stress, strong community connection may paradoxically exacerbate, rather than mitigate, the harmful effects of minority stress on well-being. This complex interplay, which we conceptualize as the paradox of belonging, offers a new perspective on the function of community for multiply marginalized individuals.

This unexpected exacerbating effect can be understood through several interrelated mechanisms. A primary explanation may be the phenomenon of trauma bonding within marginalized communities. When individuals with strong community ties come together, their shared experiences of discrimination can become a central point of connection. While this can be validating, it may also create an environment where collective conversations focus on shared grievances, reinforcing a narrative of disadvantage without necessarily providing concrete resources or strategies for improving well-being. Such spaces can validate the individual’s experience but may not alleviate, and could even deepen, their distress. This aligns with recent theoretical developments questioning whether community connectedness is always a health-enhancing factor or if it can sometimes serve as a stressor itself (Frost & Meyer, 2023).

The multidimensional framework of collective identity proposed by Ashmore et al. (2004) offers a potential explanation for this paradox. Our measure of community belongingness primarily captures dimensions of affective attachment and social embeddedness. It is possible that these dimensions, when isolated from other crucial elements of collective identity—such as a shared ideology of empowerment or a collective narrative of resistance and hope—create the conditions for trauma bonding. In other words, feeling connected and embedded in a group is not, on its own, sufficient for protection. If the content and meaning of that connection center on shared grievance without a corresponding ideology of collective action, then greater belongingness may simply amplify distress. This suggests that the truly protective forms of community are those that not only connect individuals but also equip them with empowering narratives and ideologies.

This interpretation is strongly supported by a companion study (Watts & Thrasher, 2023), which found that different types of community belongingness have differential effects on mental health and well-being. In that analysis, we conceptually and methodologically separated mental health from well-being. This reflects an important theoretical distinction often overlooked in public health literature, where mental health is sometimes conflated with overall well-being (Wren-Lewis & Alexandrova, 2021). Following a more nuanced “middle position” (Wren-Lewis & Alexandrova, 2021), our companion study operationalized mental health as the presence of negative symptomatology (i.e., depression and anxiety)—a concept distinct from illness but not identical to flourishing—while well-being was operationalized as a distinct, positive state of flourishing (i.e., the emotional, psychological, and social well-being measured by the MHC-SF in the present study) (Keyes, 2002, 2005). This conceptual separation revealed that belongingness to the general LGBTQ+ community had no significant effect on these outcomes. In contrast, belongingness to the Black community was specifically linked to better mental health (defined as fewer negative symptoms), while belongingness to the intersectional Black LGBTQ+ community was specifically linked to higher well-being (defined as more positive flourishing) (Watts & Thrasher, 2023). Therefore, our paradoxical finding in the current study, that belonging specifically to the Black LGBTQ+ community exacerbated the impact of minority stress on that same community’s well-being, is deeply complex. It suggests that the very intersectional community essential for fostering positive well-being may, under conditions of high stress, simultaneously function as a site for the trauma bonding that amplifies distress.

Furthermore, just as the mainstream “LGBTQ+ community” is a mythical monolith that often obscures the white, cisgender, and homonormative perspectives at its center (Sadika et al., 2020), the notion of a monolithic “Black LGBTQ+ community” is also a simplification. These communities are themselves complex ecosystems with internal power dynamics, hierarchies, and potential for exclusion (McCormick & Barthelemy, 2021; Parmenter et al., 2021a). Individuals with certain identities (e.g., bisexuality) may face judgment and pressure to pick a side even from within the community, preventing them from showing up as their authentic selves (Gonzalez et al., 2021; Parmenter et al., 2021a). For many, the expectation of finding a safe haven within their community can make experiences of within-group marginalization feel even more detrimental than rejection from the broader society, as it represents a betrayal from the very space anticipated to be a source of support. This internal friction can undermine the potential benefits of belonging and may explain why those most deeply embedded in the community feel the sting of minority stress most acutely. This dynamic is well-documented in recent literature, which describes how mainstream LGBTQ+ spaces often enforce a homonormativity that centers the experiences of white, cisgender community members (Page et al., 2022; Parmenter et al., 2021a; Sadika et al., 2020). This can lead to “community policing” and “gatekeeping”, where the identities of multiply marginalized members are policed, questioned, or invalidated (McCormick & Barthelemy, 2021; Parmenter et al., 2021a). For Black LGBTQ+ individuals, this can manifest as pervasive Racialized Sexual Discrimination (RSD), or sexual racism, which functions as a significant race-based stressor within the very community sought for support (Wade & Harper, 2020).

Finally, the context of our data collection—between November 2020 and January 2021—cannot be overlooked. This period was marked by the dual pandemics of COVID-19 and a national reckoning with racial injustice following the murders of George Floyd and Breonna Taylor. This environment likely heightened the salience of racial identity and the awareness of systemic persecution, potentially amplifying the effects of trauma bonding within Black communities. For Black LGBTQ+ individuals, this period may have intensified feelings of being besieged from multiple fronts, making community spaces more focused on collective grief and anxiety, thus contributing to the exacerbating effect we observed. This context also highlights the “vulnerability in visibility” that Fields et al. (2022) discuss, in which the very act of being present in public as a multiply marginalized person—whether in protest or daily life—can expose individuals to increased stigma and harassment.

### 4.1. Community as an Agentic Response

Before discussing community as an agentic response, there requires a critique and definition of the concept of “resilience”. We reject a neoliberal, individualistic framing of resilience, which can problematically place the onus of survival on the marginalized individual rather than the oppressive system. Instead, we conceptualize resilience as a collective, political, and health-promoting process rooted in community connection and action (Hudson & Romanelli, 2020; Parmenter et al., 2021b). It is not a passive trait but the active, agentic process of spacemaking (Bartlett et al., 2022), collective coping (Lockett et al., 2023), and resistance (Page et al., 2022).

Despite these risks, our finding that community belongingness has a strong, direct positive effect on well-being highlights its profound importance. In the face of exclusion from both mainstream Black and mainstream LGBTQ+ spaces, Black LGBTQ+ individuals do not remain passive victims; they engage in active, agentic community creation. This process can be understood as an act of resistance and making space—intentionally building intersectional communities to counter exclusionary structures and facilitate belonging (Page et al., 2022; Parmenter et al., 2021b). These self-created communities often take the form of chosen and created families, which serve as a primary protective factor against rejection and trauma (Hailey et al., 2020), or as intentional social bubbles designed to maintain connection and safety, a strategy observed among sexual minority women of color during the COVID-19 pandemic (Riggle et al., 2021). This process of spacemaking is a therapeutic act of resilience that fosters a sense of agency and empowerment (Bartlett et al., 2022).

### 4.2. The Function of Social Capital in Building Resilience

The communities our participants described are powerful engines for the cultivation of social capital, defined as the resources afforded by social connections (Zarwell et al., 2021). Recent scholarship proposes a useful distinction between “thick trust” (deep, internal, bonding social capital) and “thin trust” (connections to external resources and institutions) (Hwahng et al., 2022). The supportive bubbles and chosen families described in the literature are clear examples of building thick trust, a secure foundation of mutual support, reciprocity, and shared understanding among peers. The creation of this bonding capital is not an end in itself; it serves a crucial function. This foundation of thick trust empowers community members to build thin trust by collectively navigating and accessing external resources, such as affirming health care, housing, and legal services (Hwahng et al., 2022; Moore et al., 2021; Sherman et al., 2022). This aligns with findings that community connectedness is a key pathway through which social and political engagement translates into improved well-being (Roberts & Christens, 2021).

The finding that socioeconomic factors did not significantly predict well-being, while minority stress and community factors did, challenges assumptions that material resources alone can buffer against the profound psychological impacts of systemic discrimination for marginalized populations. Also, our sample’s positive correlation between age and well-being suggests a developmental trajectory of resilience. This aligns with life course perspectives that emphasize the accumulation of adaptive resources and coping strategies over time (Elder & Shanahan, 2007). Older Black LGBTQ+ adults may have had more opportunities to develop effective mechanisms for navigating minority stress, cultivate stable social support networks, and achieve a greater sense of identity integration (Kum, 2017). 

The generational finding warrants a deeper, more critical exploration, as the mechanisms of resilience may differ across cohorts. For instance, the positive association between age and well-being could reflect the accumulation of coping strategies over a lifetime (Elder & Shanahan, 2007). Older Black LGBTQ+ adults may have developed robust mechanisms for navigating a world that was overtly hostile (Kum, 2017). Conversely, younger generations, while emerging from highly stressful contexts of anti-LGBTQ and racialized victimization in school environments (Truong et al., 2020), may also possess a different framework of expectations regarding their rights to representation and community safety. This heightened awareness, combined with a narrative of disadvantage that can permeate community spaces, could potentially make experiences of exclusion and microaggression feel more acute or jarring. This generational tension between accumulated resilience and evolving expectations is a critical area for future research.

### 4.3. Limitations

Several limitations should be considered when interpreting these findings. First, the cross-sectional design precludes causal inferences about the relationships between variables. While theoretical frameworks suggest that minority stress precedes reduced well-being, and community belongingness serves as a buffer, longitudinal studies are needed to confirm these temporal relationships. Therefore, longitudinal research is crucial for tracking the development of community belongingness over time and establishing the causal direction of its relationship with mental and physical health outcomes. Second, data collection during the COVID-19 pandemic may have affected participants’ experiences of community connection, potentially underestimating the strength of the protective relationship under normal circumstances. Third, while our sample included diverse sexual and gender identities, some groups (particularly transgender women) were underrepresented, limiting our ability to examine potential within-group differences. Fourth, our use of purposive and snowball sampling, while necessary to reach such hidden populations, may have resulted in selection bias toward individuals more connected to community networks. Fifth, qualitative studies are needed to identify the specific mechanisms through which community belongingness protects against minority stress, examining, for instance, the role of shared narratives, collective action, and identity affirmation (Tropp & Wright, 2001). Sixth, while we use the concept of trauma bonding as a potential explanation for our findings, our study does not deeply theorize “trauma” itself. Future research (e.g., particularly qualitative inquiry) should explore the complex, and likely generation-specific, ways in which Black LGBTQ+ individuals define, perceive, and process trauma. Understanding the nuances of racialized and anti-LGBTQ trauma (Hailey et al., 2020) is essential for unpacking how and why it is shared within community spaces and when that sharing becomes deleterious rather than healing. Seventh, our analysis focused on the intersections of race, sexual orientation, and gender identity; we did not, however, collect data on other salient identities that are known to profoundly shape experiences of community and health, such as disability status, religion/spirituality, or immigration status. These additional layers of identity undoubtedly interact with minority stress and belonging and should be a focus of future intersectional research.

Lastly, the multidimensional framework proposed by Ashmore et al. (2004) also provides a helpful lens for understanding the limitations of this research and its future directions. While our study focused primarily on attachment, social embeddedness, and, implicitly, the content/meaning aspects of community belongingness, other dimensions, such as self-categorization and evaluation (both private and public), were not directly assessed. Future research could explore how these additional dimensions contribute to or interact with the buffering effect of community belongingness. For example, individuals who strongly self-categorize as Black LGBTQ+ and hold positive evaluations of this identity may derive even greater benefits from community connection (Sellers et al., 1997). Considering the full spectrum of collective identity dimensions could lead to more targeted and effective interventions, explain more of the variance in well-being outcomes, and further strengthen our ability to use our findings in applied settings. Additionally, our operationalization of minority stress did not distinguish between distal stressors (i.e., objective prejudice events) and proximal stressors (i.e., internalized stigma, expectations of rejection), a key distinction in minority stress theory (Meyer, 2003). Future research should measure these distinct processes to explore whether community belongingness interacts differently with external events versus internal appraisals of stigma.

### 4.4. Public Health Implications

Our findings have significant and nuanced implications for public health. The strong, direct, and positive effect of community belongingness on well-being confirms that fostering connection remains a vital public health goal. However, our moderation findings demand a more sophisticated approach. Public health interventions must move beyond simply creating spaces and instead focus on supporting the active, agentic process of spacemaking, creating venues where marginalized groups can heal, organize, and build resilience (Bartlett et al., 2022). This could include funding for Black LGBTQ+-led organizations to develop and host facilitated support groups that are resource-oriented, focusing on skill-building and collective coping strategies rather than solely on shared grievances. Mental health programs should incorporate community-building components that actively address and work to dismantle within-group power dynamics and ‘isms’, ensuring that all members, regardless of their specific gender or sexual identity, feel safe and affirmed. Importantly, these initiatives should be designed to cultivate multiple forms of social capital. This includes fostering bonding social capital (thick trust) through peer support, while also intentionally building bridging and linking social capital (thin trust) that connects community members to external resources and institutions, which is essential for navigating systemic barriers (Bartlett et al., 2022; Hwahng et al., 2022).

Within health care settings, trauma-informed and culturally responsive practices are essential. This includes training health care providers to recognize and address the specific forms of minority stress experienced by Black LGBTQ+ individuals, such as microaggressions related to their marginalized identities. Mental health programs should incorporate community-building components rather than focusing exclusively on individual therapy. This could involve reframing community engagement and collective action as legitimate therapeutic tools that can foster a virtuous cycle of individual healing and social change (Bartlett et al., 2022; Hudson & Romanelli, 2020). Further, clinical interventions can focus on cultivating internal psychological resources, such as hope, which research suggests to be a significant protective factor against the harms of minority stress (Moe et al., 2023). Such approaches could include facilitating peer support groups, connecting patients with Black LGBTQ+-affirming religious or spiritual organizations, or partnering with community-based organizations to provide holistic care.

Funding initiatives must prioritize Black LGBTQ+-led organizations, which often operate with limited resources despite delivering critical support services, culturally relevant support, advocacy, and a sense of shared identity that is frequently lacking in mainstream LGBTQ+ or Black community organizations. These entities should be understood not as optional amenities but as essential support infrastructure, the loss of which can be devastating for community health, particularly during public health crises like the COVID-19 pandemic (Konnoth, 2020). Funding should support direct services and capacity-building efforts to ensure the long-term sustainability of these vital community resources.

At the policy level, a two-pronged approach is needed. First, comprehensive anti-discrimination protections are essential to address the sources of minority stress. These protections must explicitly include both sexual and gender identity, and they must be enforced across all sectors, including housing, employment, health care, and public accommodations. Second, policies should proactively support the development and maintenance of community infrastructure that fosters a sense of belonging for Black LGBTQ+ individuals. This could include zoning regulations that facilitate establishing LGBTQ+-affirming businesses and community centers, funding for community-led initiatives, and policies protecting the right to gather and organize.

To translate these findings into effective interventions, future research must prioritize community-engaged approaches, specifically those that center the lived experiences and expertise of Black LGBTQ+ individuals. For example, community-based participatory research (CBPR) methods are well-suited for this task (Ricks et al., 2022). CBPR emphasizes equitable partnerships between researchers and community members throughout the research process, from defining research questions and designing methodologies to disseminating findings and implementing interventions (Wallerstein et al., 2017). However, this work must be undertaken with a critical awareness of the challenges to its implementation. A recent review of CBPR with SGM communities found that true, equitable partnership is rare (Blumenthal, 2011; Ricks et al., 2022), with community involvement often limited to recruitment rather than shared leadership (Ricks et al., 2022). Therefore, truly actively partnering with Black LGBTQ+ communities in the development of research and interventions can lead to more effective and sustainable solutions that promote resilience and well-being (Barr et al., 2016; Hudson & Romanelli, 2020; Lockett et al., 2023).

## 5. Conclusions

This study offers a crucial, albeit complex, message of both challenge and resilience within the Black LGBTQ+ community. While the negative impact of minority stress is undeniable, the power of the community to directly enhance well-being is equally significant. Our findings caution against a simplistic view of community as a universal panacea. Instead, they highlight that the very spaces sought for refuge can sometimes become echo chambers of distress, particularly when external societal pressures are high. The path to health equity, therefore, lies not only in fostering community but in actively building communities that are resilient, resourceful, and capable of transforming shared pain into collective power. By dismantling the systemic barriers that create minority stress and by investing in the health of community spaces themselves, we can help promote the flourishing and well-being of Black LGBTQ+ individuals.

## Figures and Tables

**Table 1:** Sample Demographics (N = 345).

Characteristic	No. (%) or Mean ± SD
**Age**	27.95 ± 6.37
**Ethnicity**	
African	20 (5.8)
Afro-Caribbean/African Caribbean	47 (13.6)
Afro-American/African American	172 (49.9)
Black	94 (27.2)
Black African	12 (3.5)
**Gender identity**	
Woman	66 (19.1)
Man	118 (34.2)
Gender-nonconforming/non-binary	81 (23.5)
Gender queer	24 (7.0)
Transgender man	28 (8.1)
Transgender woman	16 (4.6)
Two-spirit	15 (4.3)
**Sexual identity**	
Asexual	34 (9.9)
Bisexual	103 (29.9)
Down Low or DL	11 (3.2)
Gay	115 (33.3)
In the life	15 (4.3)
Lesbian	40 (11.6)
Pansexual	46 (13.3)
Queer	52 (15.1)
Same gender loving	15 (4.3)
Straight/Heterosexual	6 (1.7)
**Income ($)**	
54,999 or less	182 (56.5)
55,000 or more	140 (43.4)
**Education**	
Less than high school	36 (11.2)
High school diploma or GED	60 (18.6)
Some college, no degree	37 (11.5)
Trade school	15 (4.7)
Associates degree	17 (5.3)
Bachelor’s degree	75 (23.3)
Some graduate/professional	24 (7.5)
Graduate/professional degree	58 (18.0)
**Employment**	
Part-time	68 (21.0)
Full-time	211 (65.1)
Retired	6 (1.7)
Self-employed	12 (3.7)
Unemployed	27 (8.3)
**Area of residence**	
Rural	40 (12.4)
Suburban	157 (48.6)
Urban	126 (39.0)

Note. Respondents were able to select multiple identities for gender identity and sexual orientation. Therefore, totals may exceed 100% and categories are not mutually exclusive.

**Table 2:** Pearson Correlations Among Study Variables (N = 345).

	1	2	3	4	5	6
1. Subjective Well-being	-					
2. Minority Stress	−0.196 ***	-				
3. Community Belongingness	0.313 ***	−0.095	-			
4. MS × CB Interaction	−0.221 ***	0.191 ***	−0.247 ***	-		
5. Age	0.188 ***	−0.079	0.170 **	−0.084	-	
6. Income	0.026	−0.033	0.068	−0.134 *	0.168 **	-
7. Education	0.029	−0.101	0.113 *	−0.002	0.313 ***	0.157 **

Note. MS × CB = Minority Stress × Community Belongingness interaction term. Income was dichotomized at $54,999. Education was dichotomized (0 = less than a college degree, 1 = college degree or more). * *p* < 0.05. ** *p* < 0.01. *** *p* < 0.001. Participants surveyed in the US 2020–2021.

**Table 3:** Multiple Linear Regression Predicting Subjective Well-being (N = 345).

Variable	b	SE	t	*p*	95% CI
Constant	49.41	2.36	20.93	0	[44.77, 54.06]
Minority Stress	−0.11	0.04	−2.81	0.005	[−0.19, −0.03]
Community Belongingness	0.43	0.09	4.84	0	[0.26, 0.61]
MS *×* CB	−0.01	0.01	−2.32	0.021	[−0.02, −0.00]
Age	0.24	0.09	2.71	0.007	[0.06, 0.41]
Education	−1.26	1.22	−1.03	0.302	[−3.67, 1.14]
Income	−0.58	1.08	−0.54	0.591	[−2.70, 1.54]

Note: R^2^ = 0.159, F(6, 338) = 10.66, *p* < 0.001. MS × CB = Minority Stress × Community Belongingness interaction term. Participants were surveyed in the United States from 2020 to 2021.

**Table 4:** Simple slope analysis of the moderating effect of community belongingness.

Community Belonging	Effect	se	t	*p*	LLCI	ULCI
−4.3887	−0.0603	0.0499	−1.2077	0.2280	−0.1584	0.0379
−1.3887	−0.0967	0.0423	−2.2827	0.0231	−0.1799	−0.0134
5.6113	−0.1816	0.0456	−3.9823	0.0001	−0.2713	−0.0919

## Data Availability

The data presented in this study are available on request from the corresponding author. The data are not publicly available due to privacy and ethical restrictions.

## Abbreviations

The following abbreviations are used in this manuscript:

CBPR	Community-Based Participatory Research
LGBTQ+	Lesbian, Gay, Bisexual, Transgender, Queer, and other Sexual and Gender Minorities
MHC-SF	Mental Health Continuum—Short Form
REDCap	Research Electronic Data Capture
RSD	Racialized Sexual Discrimination
VIF	Variance Inflation Factor

## References

[B1-behavsci-15-01604] Aiken L. S., West S. G. (1991). Multiple regression: Testing and interpreting interactions.

[B2-behavsci-15-01604] Ashmore R. D., Deaux K., McLaughlin-Volpe T. (2004). An organizing framework for collective identity: Articulation and significance of multidimensionality. Psychological Bulletin.

[B3-behavsci-15-01604] Balsam K. F., Molina Y., Beadnell B., Simoni J., Walters K. (2011). Measuring multiple minority stress: The LGBT people of color microaggressions scale. Cultural Diversity and Ethnic Minority Psychology.

[B4-behavsci-15-01604] Barr S. M., Budge S. L., Adelson J. L. (2016). Transgender community belongingness as a mediator between strength of transgender identity and well-being. Journal of Counseling Psychology.

[B5-behavsci-15-01604] Bartlett A., Faber S., Williams M., Saxberg K. (2022). Getting to the root of the problem: Supporting clients with lived-experiences of systemic discrimination. Chronic Stress.

[B6-behavsci-15-01604] Baumeister R. F., Leary M. R. (1995). The need to belong: Desire for interpersonal attachments as a fundamental human motivation. Psychological Bulletin.

[B7-behavsci-15-01604] Blumenthal D. S. (2011). Is community-based participatory research possible?. American Journal of Preventive Medicine.

[B8-behavsci-15-01604] Choi S. K., Wilson B. D. M., Mallory C. (2021). Black LGBT adults in the U.S.: LGBT well-being at the intersection of race.

[B9-behavsci-15-01604] Craney T. A., Surles J. G. (2002). Model-dependent variance inflation factor cutoff values. Quality Engineering.

[B10-behavsci-15-01604] Crenshaw K. (1989). Demarginalizing the intersection of race and sex: A Black feminist critique of antidiscrimination doctrine, feminist theory and antiracist politics. University of Chicago Legal Forum.

[B11-behavsci-15-01604] DeMarco T. C., Newheiser A.-K. (2019). When groups do not cure: Group esteem moderates the social cure effect. European Journal of Social Psychology.

[B12-behavsci-15-01604] Diener E., Oishi S., Tay L. (2018). Advances in subjective well-being research. Nature Human Behaviour.

[B13-behavsci-15-01604] Elder G. H., Shanahan M. J., Damon W., Lerner R. M. (2007). The life course and human development. Handbook of child psychology.

[B14-behavsci-15-01604] English D., Carter J. A., Boone C. A., Forbes N., Bowleg L., Malebranche D. J., Talan A. J., Rendina H. J. (2021). Intersecting structural oppression and Black sexual minority men’s health. American Journal of Preventive Medicine.

[B15-behavsci-15-01604] Fields E. L., Long A., Silvestri F., Bademosi K., Benton-Denny J., Granderson R., Schumacher C., Chandran A., Greenbaum A., Jennings J. (2022). #ProjectPresence: Highlighting black LGBTQ persons and communities to reduce stigma: A program evaluation. Evaluation and Program Planning.

[B16-behavsci-15-01604] Frost D. M., Meyer I. H. (2023). Minority stress theory: Application, critique, and continued relevance. Current Opinion in Psychology.

[B17-behavsci-15-01604] Gonzalez K. A., Flanders C. E., Pulice-Farrow L., Bartnik A. (2021). “It’s almost like bis, pans kind of stick together:” Bi+ belonging and community connection. Journal of Bisexuality.

[B18-behavsci-15-01604] Hailey J., Burton W., Arscott J. (2020). We are family: Chosen and created families as a protective factor against racialized trauma and anti-LGBTQ oppression among African American sexual and gender minority youth. Journal of GLBT Family Studies.

[B19-behavsci-15-01604] Harris P. A., Taylor R., Thielke R., Payne J., Gonzalez N., Conde J. G. (2009). Research Electronic Data Capture (REDCap)—A metadata-driven methodology and workflow process for providing translational research informatics support. Journal of Biomedical Informatics.

[B20-behavsci-15-01604] Hudson K. D., Romanelli M. (2020). “We are powerful people”: Health-promoting strengths of LGBTQ communities of color. Qualitative Health Research.

[B21-behavsci-15-01604] Husbands W., Lawson D. O., Etowa E. B., Mbuagbaw L., Baidoobonso S., Tharao W., Yaya S., Nelson L. E., Aden M., Etowa J. (2022). Black Canadians’ exposure to everyday racism: Implications for health system access and health promotion among urban Black communities. Journal of Urban Health.

[B22-behavsci-15-01604] Hwahng S. J., Allen B., Zadoretzky C., Doucet H. B., McKnight C., Des Jarlais D. (2022). Thick trust, thin trust, social capital, and health outcomes among trans women of color in New York City. International Journal of Transgender Health.

[B23-behavsci-15-01604] Institute of Medicine (2011). The health of lesbian, gay, bisexual, and transgender people: Building a foundation for better understanding.

[B24-behavsci-15-01604] Keyes C. L. M. (2002). The mental health continuum: From languishing to flourishing in life. Journal of Health and Social Behavior.

[B25-behavsci-15-01604] Keyes C. L. M. (2005). Mental illness and/or mental health? Investigating axioms of the complete state model of health. Journal of Consulting and Clinical Psychology.

[B26-behavsci-15-01604] Konnoth C. J., Burris S., de Guia S., Gable K. L. C., Levin D. E., Parmet W. E., Terry N. P. (2020). Supporting LGBT communities in the COVID-19 pandemic. Assessing legal responses to COVID-19.

[B27-behavsci-15-01604] Kum S. (2017). Gay, gray, black, and blue: An examination of some of the challenges faced by older LGBTQ people of color. Journal of Gay & Lesbian Mental Health.

[B28-behavsci-15-01604] Lockett G. M., Klein K. G., Mike J., Sostre J. P., Abreu R. L. (2023). “To feel supported in your community is to feel loved”: Cultivating community and support for Black transmasculine people navigating anti-Black racism, transphobia, and COVID-19 pandemic. International Journal of Transgender Health.

[B29-behavsci-15-01604] McConnell E. A., Janulis P., Phillips G., Truong R., Birkett M. (2018). Multiple minority stress and LGBT community resilience among sexual minority men. Psychology of Sexual Orientation and Gender Diversity.

[B30-behavsci-15-01604] McCormick M., Barthelemy R. S. (2021). Excluded from “inclusive” communities: LGBTQ youths’ perception of “their” community. Journal of Gay & Lesbian Social Services.

[B31-behavsci-15-01604] Meyer I. H. (1995). Minority stress and mental health in gay men. Journal of Health and Social Behavior.

[B32-behavsci-15-01604] Meyer I. H. (2003). Prejudice, social stress, and mental health in lesbian, gay, and bisexual populations: Conceptual issues and research evidence. Psychological Bulletin.

[B33-behavsci-15-01604] Moe J., Sparkman-Key N., Gantt-Howrey A., Augustine B., Clark M. (2023). Exploring the relationships between hope, minority stress, and suicidal behavior across diverse LGBTQ populations. Journal of LGBTQ Issues in Counseling.

[B34-behavsci-15-01604] Moore K., Camacho D., Spencer-Suarez K. N. (2021). A mixed-methods study of social identities in mental health care among LGBTQ young adults of color. American Journal of Orthopsychiatry.

[B35-behavsci-15-01604] Page K. V., Cerezo A., Ross A. (2022). Creating space for ourselves: Black sexual minority women and gender diverse individuals countering anti-Black racism and heterosexism. Psychology of Sexual Orientation and Gender Diversity.

[B36-behavsci-15-01604] Parmenter J. G., Galliher R. V., Maughan A. D. A. (2021a). LGBTQ+ emerging adults perceptions of discrimination and exclusion within the LGBTQ+ community. Psychology & Sexuality.

[B37-behavsci-15-01604] Parmenter J. G., Galliher R. V., Wong E., Perez D. (2021b). An intersectional approach to understanding LGBTQ+ people of color’s access to LGBTQ+ community resilience. Journal of Counseling Psychology.

[B38-behavsci-15-01604] Quinn K. G., Dickson-Gomez J., Craig A., John S. A., Walsh J. L. (2023). Intersectional discrimination and PrEP use among young Black sexual minority individuals: The importance of Black LGBTQ communities and social support. AIDS and Behavior.

[B39-behavsci-15-01604] Ricks J. M., Arthur E. K., Stryker S. D., Yockey R. A., Anderson A. M., Allensworth-Davies D. (2022). A systematic literature review of community-based participatory health research with sexual and gender minority communities. Health Equity.

[B40-behavsci-15-01604] Riggle E. D. B., Drabble L. A., Bochicchio L. A., Wootton A. R., Veldhuis C. B., Munroe C., Hughes T. L. (2021). Experiences of the COVID-19 pandemic among African American, Latinx, and White sexual minority women: A descriptive phenomenological study. Psychology of Sexual Orientation and Gender Diversity.

[B41-behavsci-15-01604] Roberts L. M., Christens B. D. (2021). Pathways to well-being among LGBT adults: Sociopolitical involvement, family support, outness, and community connectedness with race/ethnicity as a moderator. American Journal of Community Psychology.

[B42-behavsci-15-01604] Rosenberg R. D. (2021). Negotiating racialised (un)belonging: Black LGBTQ resistance in Toronto’s gay village. Urban Studies.

[B43-behavsci-15-01604] Sadika B., Wiebe E., Morrison M. A., Morrison T. G. (2020). Intersectional microaggressions and social support for LGBTQ persons of color: A systematic review of the Canadian-based empirical literature. Journal of GLBT Family Studies.

[B44-behavsci-15-01604] Sellers R. M., Rowley S. A., Chavous T. M., Shelton J. N., Smith M. A. (1997). The multidimensional inventory of black identity: A preliminary investigation of reliability and construct validity. Journal of Personality and Social Psychology.

[B45-behavsci-15-01604] Sherman A. D. F., Allgood S., Alexander K. A., Klepper M., Balthazar M. S., Hill M., Cannon C. M., Dunn D., Poteat T., Campbell J. (2022). Transgender and gender diverse community connection, help-seeking, and mental health among Black transgender women who have survived violence: A mixed-methods analysis. Violence Against Women.

[B46-behavsci-15-01604] Tropp L. R., Wright S. C. (2001). Ingroup identification as the inclusion of ingroup in the self. Personality and Social Psychology Bulletin.

[B47-behavsci-15-01604] Truong N. L., Zongrone A. D., Kosciw J. G. (2020). Erasure and resilience: The experiences of LGBTQ students of color, Black LGBTQ youth in U.S. schools.

[B48-behavsci-15-01604] Wade R. M., Harper G. W. (2020). Racialized Sexual Discrimination (RSD) in the age of online sexual networking: Are young Black gay/bisexual men (YBGBM) at elevated risk for adverse psychological health?. American Journal of Community Psychology.

[B49-behavsci-15-01604] Wallerstein N., Duran B., Oetzel J., Minkler M. (2017). Community-based participatory research for health: From process to outcomes.

[B50-behavsci-15-01604] Watts K. J., Thrasher S. S. (2023). The impact of community belongingness on mental health and well-being among Black LGBTQ adults. Race and Social Problems.

[B51-behavsci-15-01604] Wren-Lewis S., Alexandrova A. (2021). Mental health without well-being. Journal of Medicine and Philosophy.

[B52-behavsci-15-01604] Zarwell M., Walsh J. L., Quinn K. G., Kaniuka A., Patton A., Robinson W. T., Cramer R. J. (2021). A psychometric assessment of a network social capital scale among sexual minority men and gender minority individuals. BMC Public Health.

